# Structural determinants for binding to angiotensin converting enzyme 2 (ACE2) and angiotensin receptors 1 and 2

**DOI:** 10.3389/fphar.2015.00005

**Published:** 2015-01-30

**Authors:** Daniel Clayton, Iresha Hanchapola, Walter G. Thomas, Robert E. Widdop, Alexander I. Smith, Patrick Perlmutter, Marie-Isabel Aguilar

**Affiliations:** ^1^Department of Biochemistry and Molecular Biology, Monash UniversityClayton, VIC, Australia; ^2^School of Biomedical Sciences, University of QueenslandBrisbane, QLD, Australia; ^3^Department of Pharmacology, Monash UniversityClayton, VIC, Australia; ^4^School of Chemistry, Monash UniversityClayton, VIC, Australia

**Keywords:** angiotensin II, angiotensin II receptor 1, angiotensin II receptor 2, angiotensin converting enzyme-2, β-amino acids

## Abstract

Angiotensin converting enzyme 2 (ACE2) is a zinc carboxypeptidase involved in the renin–angiotensin system (RAS) and inactivates the potent vasopressive peptide angiotensin II (Ang II) by removing the C-terminal phenylalanine residue to yield Ang1–7. This conversion inactivates the vasoconstrictive action of Ang II and yields a peptide that acts as a vasodilatory molecule at the Mas receptor and potentially other receptors. Given the growing complexity of RAS and level of cross-talk between ligands and their corresponding enzymes and receptors, the design of molecules with selectivity for the major RAS binding partners to control cardiovascular tone is an on-going challenge. In previous studies we used single β-amino acid substitutions to modulate the structure of Ang II and its selectivity for ACE2, AT_1_R, and angiotensin type 2 (AT_2_R) receptor. We showed that modification at the C-terminus of Ang II generally resulted in more pronounced changes to secondary structure and ligand binding, and here, we further explore this region for the potential to modulate ligand specificity. In this study, (1) a library of 47 peptides derived from the C-terminal tetrapeptide sequence (-IHPF) of Ang II was synthesized and assessed for ACE2 binding, (2) the terminal group requirements for high affinity ACE2 binding were explored by and N- and C-terminal modification, (3) high affinity ACE2 binding chimeric AngII analogs were then synthesized and assessed, (4) the structure of the full-length Ang II analogs were assessed by circular dichroism, and (5) the Ang II analogs were assessed for AT_1_R/AT_2_R selectivity by cell-based assays. Studies on the C-terminus of Ang II demonstrated varied specificity at different residue positions for ACE2 binding and four Ang II chimeric peptides were identified as selective ligands for the AT_2_ receptor. Overall, these results provide insight into the residue and structural requirements for ACE2 binding and angiotensin receptor selectivity.

## INTRODUCTION

Angiotensin II is the central active component of the RAS and signals primarily through the AT_1_R, and production of Ang II is catalyzed by the degradation of angiotensin by ACE. High blood pressure, or hypertension, is the main risk factor of cardiovascular disease and so two major therapeutic targets for treatment of hypertension are ACE and the AT_1_R. Although there are a number of anti-hypertensive drugs on the market such as ACE inhibitors (e.g., Captopril, Enalapril), and AT_1_R antagonists/blockers (e.g., Losartan, Valsartan) individual responses and side-effect profiles are highly variable, and often a mixed therapeutic regime is necessary. This is largely attributable to the complexity of RAS, the level of cross-talk between key molecules such as AngII, its cleavage products, and their binding partners [e.g., ACE2, AT_1_R, AT_2_R, Mas receptor (MasR, Santos et al., 2003)] and the resulting activation of pathways downstream of receptor binding.

Angiotensin converting enzyme 2, a zinc carboxypeptidase (Donoghue et al., 2000; Tipnis et al., 2000; Turner et al., 2002), the AT_1_R and the AT_2_R and their Ang II-derived ligands are all involved in modulating hypertension (Donoghue et al., 2000; Tipnis et al., 2000; Turner et al., 2002; Jiang et al., 2014). ACE2 is also up-regulated in the heart in human and animal models of cardiovascular disease and specifically in the human fibrotic liver [ref]. Ang1–7 is generated by the action of ACE2 (Vickers et al., 2002) and is now considered a major component of RAS and has been shown to exert specific actions via binding to MasR (Santos et al., 2003, 2008; Bader et al., 2014). A number of compounds have been developed that inhibit ACE2 activity [DX600/MLN4760, Millennium Pharmaceuticals, Cambridge, MA, USA (Dales et al., 2002)], or activate activity (Hernandez Prada et al., 2008). Some of these ACE2 inhibitors have been shown to increase cardiac hypertrophy and fibrosis (Trask et al., 2010) while ACE2 activators have been shown to have significant anti-hypertensive action and be effective in the reversal of cardiac and renal fibrosis (Varagic et al., 2014). Given the central role of ACE2 in inactivating Ang II and generating Ang1–7, a more detailed description of the molecular specificity of AngII–ACE2 interaction is important. Knowledge on how Ang II analogs with altered ACE2 binding specificity act at the AT_1_R and AT_2_R is useful to identify compounds with selective action for potential therapeutic use.

We previously described the influence of single β-amino acid substitutions on the structure and binding of Ang II to ACE2 (Clayton et al., 2011). We demonstrated that three different regions of Ang II that exert different effects on Ang II structure and binding, namely the N-terminus, the central and the C-terminal region. We also showed that the β-turn conformation is the structural determinant for enhanced substrate cleavage. In this previous study the C-terminus was the most susceptible to changes and in the present study we have further explored the structural requirements of this region of Ang II in regards to ACE2 and AT receptor binding. We hypothesized that the C-terminus of Ang II is the main binding determinant for ACE2, and here we further explore this specificity, and also the specificity of Ang II for its other binding partners (AT_1_R and AT_2_R) by residue substitution in the C-terminus of Ang II. The structural requisites for binding to these different protein targets were also explored by circular dichroism (CD) analysis of the Ang II analogs.

## MATERIALS AND METHODS

### CHEMICALS

General peptide synthesis reagents were purchased from GL Biochem (Shanghai, China) and AusPep (Melbourne, VIC, Australia), high purity acetonitrile from Merck (Whitehouse Station, NJ, USA), and high purity formic acid for LC–MS from Fluka (Sigma-Aldrich, St. Louis, MO, USA). β-amino acids were from Peptech (Burlington, MA, USA) and the ACE2 QFS from AusPep.

### PEPTIDE SYNTHESIS

Angiotensin II, β-substituted and C-terminal tetrapeptide analogs were prepared using Fmoc solid-phase peptide synthesis (SPPS) approaches on Wang resin using standard side chain protecting groups. Generally, a 3-fold excess of amino acid and HATU, and 4.5-fold excess of DIPEA with a coupling time of 30 min was used for manual synthesis. The extent of the amino acid couplings was monitored using the ninhydrin reaction (Sarin et al., 1981). Amidated Ang II tetrapeptides were made on Rink Amide resin (Novabiochem/Merck-Millipore) and N-terminal acetylation was performed for 10 min with a 10% acetic anhydride, 2% DIPEA, DMF solution. Microwave-assisted peptide synthesis was performed on a CEM Liberty system (USA, NC) using a 5-fold excess of amino acid and HBTU with 5 min irradiated coupling times. Peptides were cleaved from the resin and deprotected with a TFA cleavage solution (TFA/TIPS/H_2_O, 95:2.5:2.5), for 2 h at room temperature.

### HPLC PURIFICATION AND ANALYSIS

Angiotensin II analogs were purified by RP–HPLC from crude peptide, on an Agilent (Agilent Technologies, Palo Alto, CA, USA) HP1200 system using a Vydac, 10 × 250 mm C4 column. Linear gradients were from 10 to 25% acetonitrile (0.1% TFA). All peptides were identified by MS an MSD VL3000 ion-trap mass spectrometer (Agilent Technologies, Palo Alto, CA, USA). Purity of the peptides was determined by RP–HPLC on a 150 × 4.6 mm C18 column. Ang II analogs were exchanged to the chloride salt for QFS assays by re-dissolving them in a 15-fold excess of aqueous HCl, 10 min standing, followed by lyophilization. Peptides were then re-dissolved in 50% acetonitrile/water and re-lyophilized to yield a white powder.

### ACE2 QUENCHED FLUORESCENCE SUBSTRATE (QFS) ASSAYS

Angiotensin converting enzyme 2 QFS assays were performed for 1 h in 50 uM QFS, 1 M NaCl, 100 mM Tris-Cl, pH 6.5 at 37∘C on a BMG LabTechnologies (Offenburg, Germany), FLUOStar Optima in a 96 well-plate. Subsequent data analysis determined the inhibition of native AngII, its analogs and C-terminal peptides compared to the ACE2 QFS substrate [MCA-APK(DNP)-OH, Vickers et al., 2002]. Initially, analogs were screened in triplicate at 100 μM and those showing significant inhibition as compared to control peptides, either AngII (DRVYIHPF), or the C-terminal IHPF, were then assayed at concentrations of 10 μM or lower. Three controls were included in each ACE2 QFS assay; AngII (100 and 10 μM), IHPF (100 μM) and a known ACE2 inhibitor (1 nM; Millennium Pharmaceuticals, Cambridge, MA, USA). QFS assay results were accepted when ACE2 inhibition by AngII (10 μM) was 80 ± 5%, IHPF was 65 ± –5% and the (Qian et al., 1999) ‘Millennium Inhibitor’ was 70 ± –5%. Acceptable coefficients of variation (CV; standard deviation/mean × 100) for sample triplicates were <7%.

### CIRCULAR DICHROISM

Circular dichroism measurements were performed on a Jasco J-810 Circular Dichroism Spectropolariser (Jasco, Tokyo, Japan) using quartz cuvettes of 1 mm path length. Scans between 190 and 260 nm were performed at a scan speed of 20 nm/min, bandwidth of 1.0 nm, resolution of 0.1 nm, a 1 s response time and with three scan accumulations. The quartz cuvette temperature was controlled (25∘C) with a Peltier temperature controller and the CD instrument was calibrated with (+)-10-camphorsulfonic acid. CD spectra of Ang II and β-analogs were measured in /10 mM phosphate buffer at 7.0, and spectra were smoothed using the Jasco Fast Fourier transform algorithm and then baseline corrected. Peptide concentration was approximately 200 μM for each analog. The concentration of each solution was normalized by peak integration at 214 nm by RP–HPLC to ensure similar concentration for each peptide solution. Furthermore, the CD spectra of the individual aromatic amino acids present in Ang II were recorded free in solution at similar concentration and then subtracted from the spectra of the Ang II analogs to minimize the contaminating CD signal from aromatic side chains.****

### LC–MS ACE2 PROTEOLYTIC CLEAVAGE ASSAY

Angiotensin II analogs (10 μM) were incubated at 37∘C in the presence of ACE2 until over a half of the Ang II control was cleaved (as determined by LC–MS), typically between 4 and 5 h then proteolysis was quenched by the addition of 40 μl of 4 M urea/50% acetonitrile/0.2% trifluoroacetic acid. The first hour of the incubation was performed in the Fluorostar Optima plate reader in order to confirm previously observed level of inhibition, then for the remaining time the samples were transferred to small microfuge tubes and incubated in a water bath. The extent of ACE2 cleavage was assessed by LC–MS on an Agilent capillary HP1100, MSD VL3000 Ion-Trap system.

### ANGIOTENSIN RECEPTOR BINDING EXPERIMENTS

The generation of plasmids expressing HA-tagged versions of the AT1R and AT2R have been previously described Qian et al. (1999), D’Amore et al. (2005). HEK-293 cells in 12 well-plates were transfected with either AT1R or AT2R plasmids (0.6 μg/well) using lipofectAMINE (4⋅8 μl/well), as previously described Thomas et al. (1998) and stably expressing clones obtained by selection with G418 (1 mg/ml) and limiting dilution. HEK clones expressing either AT1R or AT2R were plated in 12 well-plates for whole cell competition binding assays using the non-selective Ang II ligand, [^125^I]-Ang II and selected concentrations of Ang II, PD123319 (an AT2-selective ligand) and the various β-substituted Ang II peptides. Non-linear regression of the data was achieved using GraphPad Prism (GraphPad Software Inc., San Diego, CA, USA) to determine relative displacement of chimeric Ang II analogs as compared to ^125^I-Ang II in either AT1R- or AT2R- transfected HEK-293 cells.

## RESULTS

### Ang II-ACE2 BINDING REQUIREMENTS

A library of 40 tetrapeptides based on the four C-terminal residues of Ang II (Ile-His-Pro-Phe/IHPF) was generated and screened for ACE2 binding at 100 μM in order to identify key residues of the C-terminus of Ang II involved in binding to the ACE2 active site. Peptide sequences and the apparent binding values are listed in **Table 1**. ACE2 binding was assessed indirectly by use of a QFS assay where the ability of the compound to reduce the proteolytic cleavage of a fluorogenic substrate shows relative ACE2 binding (Clayton et al., 2011). Several substitutions (Val, Tyr, Ala, Gly, Phe, Trp, Pro, His, Ile, and Leu) were made in order to further probe the highly hydrophobic and cyclic specificity of the C-terminus of Ang II. A β-napthalene amino acid (Nth) derivative was used as a highly hydrophobic non-natural cyclic residue substitute, and Arg and Lys were substituted for histidine at the second position from the N-terminus of the tetrapeptide to determine if positive charge or other structural or positional requirements were important.

**Table 1:** Angiotensin converting enzyme 2 inhibition of single substitution analogs of the C-terminal of AngII^**a,b,c,d,e**^.

Substitution (X)	XHPF	IXPF	IHXF	IHPX
AngII C-terminal – IHPF				
Valine (V)	78	28	77	18
Tyrosine (Y)	34	87	1	50
Alanine (A)	59	37	19	44
Glycine (G)	0	39	0	41
Phenylalanine (F)	48	0	0	
Tryptophan (W)	0	40	0	57
Proline (P)	78	0	–	*
Histidine (H)	12	–	14	54
Isoleucine (I)	–	32	44	36
Leucine (L)	–	–	0	48
Napthaline (Npth)	18	39	53	55
Lysine (K)	–	28	–	–
Arginine (R)	–	33	–	–

*Unmodified Angiotensin II sequence – H_*2*_N- 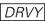 - 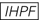 -COO(-)*.

^a^The position of the substitution is indicated as ‘X’ and in bold at top of column, ^b^peptides screened at 100 μM, ^c^native IHPF C-terminal shows 62% Inh. at 100 μM, ^d^napthaline represents a β-napthalene amino acid analog and, ^e^slightly negative values observed have been assigned as 0% inhibition. *Synthesis of IHPP was attempted twice but unsuccessful.

The requirements for ACE2 binding at the first position of the tetrapeptide [fourth position from the Ang II C-terminus (XHPF)] were a preference for non-polar, hydrophobic or cyclic residues, with Val and Pro substitutions showing enhanced binding [both 78% inhibition (Inh.)], and Ala showing similar binding (59% Inh.) compared to the native C-terminal of Ang II (IHPF, 62% Inh.). Large bulky functional groups were less tolerated at this position with substantial reductions in ACE2 binding observed when tryptophan or a naphthalene-derived amino acid was placed at this position (0, 18% Inh.). Interestingly, substitution of the minimal amino acid residue Gly to this position abolished ACE2 binding.

No strict preference was observed at position two of the tetrapeptide (IXPF, the third position from the C-terminus), with a range of analogs able to bind ACE2 and inhibit QFS cleavage (Val, Ala, Gly, Trp, Ile, and Nth, 28–40% Inh.). The two residues not tolerated at this position were the apolar cyclic residues Phe and Pro, which showed no ACE2 binding at 100 μM. Interestingly, when another hydrogen bond donating side chain, tyrosine, was substituted at this position, ACE2 binding was enhanced (IYPF 87%, IHPF 62% Inh.). ACE2 binding was completely abolished by substitution of either Phe or Pro to this position (0% Inh.) while substitution of the His for other positively charged residues also resulted in moderate reductions in ACE binding (Arg 33%, Lys 28% Inh.).

The most stringent side chain requirements were seen for the third position of the tetrapeptide (IHXF, the second position from the C-terminus), or the P1’ site of the scissile-bond with only three out of the ten substitutions tolerated. Substitution of Val or Ile to this position resulted in either moderate increases (Val, 77% Inh.) or decreases (Ile, 44% Inh.) to ACE2 binding. The other functional group tolerated at this position was napthalene with this analog showing a slight decrease in ACE2 binding (53% Inh.). The observation that two of the three residues tolerated at this position are β-branched amino acids indicates some peptide structural constraint, or conferred conformation is a requirement for ACE2 binding. All other analogs at this position showed minimal or substantially reduced binding with Tyr, Gly, Phe Trp, His, Ala, or Leu showing between 0 and 19% inhibition.

Minimal specificity was observed for residues at the C-terminal residue, or at the P1 site of the scissile-bond. All substitutions at this position exhibited some level of ACE2 binding, with the largest decrease observed when Val was tested at the first residue (18% Inh.). Other substitutions, ranging from compact (Gly/Ala), bulky (Nth/Trp), apolar (Ile/Leu) to a polar (His) side chain were tolerated with only minor to moderate decreases in binding observed (36 to 57% Inh. vs. 62% IHPF).

By combining the optimal substitutions at the first, second and third positions in IHPF, seven tetrapeptide analogs were generated of which three showed almost equivalent ACE2 binding compared to the full-length native Ang II (Ang II 97% Inh., PYPF/PHVF/PYVF 93/94% Inh.; peptide sequences and binding data listed in **Table 2**). These three analogs showed almost saturating levels of inhibition at the screening concentration of 100 μm. Three further modifications were made to these seven peptides, and to the control peptide IHPF, to probe the binding requirements of the termini and assess the binding of these C-terminal analogs in the absence of a potentially shielding N-terminal positive charge; N-terminal acetylation, C-terminal amidation and, the combination of both modifications. These terminally modified peptides which are listed in **Table 3**, were first screened at 100 μM, and the peptides that retained high levels of binding were then screened at 10 μM to further evaluate ACE2 binding (**Table 3**). Although N-terminal acetylation removes the positive charge and enhances hydrophobicity, and thus was anticipated to enhance ACE2 interaction, both increases and decreases in inhibition were observed. The only clear trend was that N-capping an analog with an N-terminal Pro always resulted in decreases in ACE2 inhibition. The largest decrease in ACE2 binding was seen when the PHVF analog was acetylated (8% Inh. at 100 μM), while other N-terminal Pro analogs showed more moderate decreases. Three N-capped peptides showing increased binding were, IHPF (87% Inh. vs. 66% unmodified), IYPF (93 vs. 84% unmodified) and VYPF (98 vs. 78% unmodified), while other analogs showed minor to moderate decreases in binding (**Table 3**).

**Table 2:** Angiotensin converting enzyme 2 inhibition of multiple residue substitution analogs^**a,b,c**^.

Single substitutions	Inhibition (%)	Multiple substitutions	Inhibition (%)
From **Table 1**		**VY**PF	90
**V**HPF	78	**PY**PF	93
**P**HPF	81	**V**H **V**F	85
I **Y**PF	84	**P**H **V**F	93
IH **V**F	77	I **YV**F	77
		**VYV**F	78
		**PYV**F	94

*Unmodified Angiotensin II sequence – H_*2*_N- 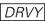 - 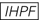 -COO(-).*

*Residue substitutions shown in underlined and in bold; ^a^peptides screened at 100 μM, ^b^multiple substitution based on optimal single residue substitutions at first, second and third position, ^c^native IHPF C-terminal shows 62% inhibition at 100 μM*.

**Table 3:** Angiotensin converting enzyme 2 inhibition of terminally modified IHPF analogs^**a**^.

AngII analog	Unmodified	*N*-acetylated	C-amidated	*N*-acetylated/C-amidated
	100 (10) μM	100 (10) μM	100 (10) μM	100 (10) μM
IHPF	66 (22)	87 (41)	18	26
**P**H**P**F	81 (20)	57	1	8
I **Y**PF	84 (35)	93 (62)	8	18
**VY**PF	78 (26)	98 (79)	25	30
**PY**PF	93 (50)	87 (44)	33	28
**V**H **V**F	85 (37)	76 (31)	86 (40)	21 (4)
**P**H **V**F	93 (49)	8	12	12
**PYV**F	94 (51)	78 (18)	26	25

*Unmodified Angiotensin II sequence is – H_*2*_N- 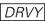 – 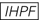 -COO(-)*.

*^a^Analogs were first screened at 100 μM, then those showing inhibition greater than IHPF (66%) were screened at 10 μM (in brackets)*.

As expected, the presence of a C-terminal carboxylate was required to maintain high levels of ACE2 binding, with moderate to large decreases (1–33% Inh.) in binding observed for all but one C-terminally amidated analogs (**Table 3**). The VHVF analog showed no change (86% Inh. vs. 85% Inh. unmodified) in ACE2 binding even though the C-terminal carboxylate is abolished, indicating a distinct mode of binding for this compound. The only amidated analog whose ACE2 binding was substantially altered by N-terminal capping was VHVF, which showed a 4-fold reduction in ACE2 inhibition by QFS assays. This indicates the N-terminal positive charge of this peptide may play a key role in binding for this analog, either through direct interaction or via structural stabilization. Assessing Ang II analogs at less saturating concentrations (10 μM) was performed to better characterize the relative ACE2 binding differences of the modified peptides (**Table 3**; 10 μM data shown in brackets). This revealed the largest increases in ACE2 binding for acetylated IYPF and acetylated VYPF, which showed a three to 4-fold overall increase in binding (62 and 79% Inh.) as indicated by QFS assays. Other peptides showed more moderate increases to ACE2 binding.

In summary, based on the results for the Ang II C-terminal analogs, significant increases in apparent binding to ACE2 were observed for substitution of isoleucine by proline or valine at the first position of IHPF, tyrosine for histidine at the second position, and valine for proline at the third position. Combinations of these favorable single residue substitutions generally resulted in further increases in apparent ACE2 binding. N-terminal acetylation of the most potent tetrapeptides resulted in increased inhibition for many peptides while C-terminal amidation greatly diminished apparent binding of all but one of these tetrapeptides, VHVF. The tetrapeptides having the highest binding to ACE2 had IC_50_ values in the 5–20 μM range (full-length AngII, IC_50_, ∼5 μM).

### ACE2 BINDING OF Ang II CHIMERAS

Chimeric Ang II analogs were prepared by combining key elements of ACE2 binding and proteolytic stability identified in previous experiments. These peptides contained the most inhibitory acetylated C-terminal tetrapeptides (**Table 3**) grafted to the native N-terminal sequence of Ang II to further enhance ACE2 binding and are listed in **Table 4**. Two β-amino acid analogs of Ang II which stabilize the scissile-bond (β-Pro^7^ and β-Phe^8^) in Ang II (Clayton et al., 2011) were also tested for comparative purposes (**Table 4**).

**Table 4:** Angiotensin converting enzyme 2 inhibition and cleavage of AngII chimeras^**a,b**^.

	100 μM	10 μM	1 μM	0.1 μM
**Inhibition (%)**
AngII (DRVYIHPF)	99 ± 0.3	77 ± 2.9	21 ± 6.8	–5 ± 8.1
DRVYI **Y**PF	–	96 ± 0	78 ± 2	17* ± 6
DRVY **VY**PF	–	98 ± 0.3	84 ± 2.7	31 ± 3.5
DRVYI **YβP**F	72 ± 1.5	10 ± 5.8	–4 ± 9.8	–
DRVY **VYβP**F	90 ± 0.6	42 ± 4.4	0 ± 11.4	–
DRVYI **Y**P**βF**	53 ± 1.9	0 ± 5	–1 ± 6.8	–
DRVY **VY**P**βF**	59 ± 2.1	15 ± 9	2 ± 3.6	–
**Cleavage (%)**
AngII (DRVYIHPF)	–	40* ± 2	93	–
DRVYI **Y**PF	–	18 ± 1.2	85	–
DRVY **VY**PF	–	9 ± 0.3	64	–
DRVYI **YβP**F	0 ± 0	0 ± 0	–	–
DRVY **VYβP**F	0 ± 0	0 ± 0	–	–
DRVYI **Y**P**βF**	0 ± 0	0 ± 0	–	–
DRVY, **VY**P**βF**	0 ± 0	0 ± 0	–	–

*Unmodified Angiotensin II sequence – H_*2*_N- 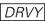 - 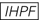 -COO(-)*.

*^a^Peptides were assayed for ACE2 inhibition at 10 and 1 μM, and for ACE2 proteolytic cleavage at 10 μM then depending on their inhibition levels at either higher or lower concentrations; ^b^ACE2 proteolytic cleavage assessed over 4 h*.

*Averages are based on two replicate values.

Enhancements to apparent ACE2 inhibition were observed when the native N-terminal sequence was combined with the most inhibitory C-terminal sequences. The Ang II chimera DRVYIYPF showed 96% inhibition at 10 μM (**Table 4**) as compared to the acetylated peptide IYPF (**Table 3**, 62% at 10 μM), and showed notably more inhibition than the native Ang II (77% at 10 μM). DRVYVYPF also showed increased levels of ACE2 inhibition compared to the capped VYPF, showing 98% inhibition (**Table 4**) as compared to 79% (**Table 3**). This Ang II chimera also exhibited increased apparent ACE2 binding compared to native Ang II which exhibited 77% inhibition at 10 μM. Significant decreases in binding were observed when β-Pro was substituted at the scissile-bond of both these Ang II chimeras; the ACE2 inhibition of DRVYIYβPF decreased to 10% while that of DRVYVYβPF decreased to 42%. Notable drops in ACE2 binding were also seen for the two β-Phe Ang II analogs with binding completely abolished in DRVYIYPβF and 15% inhibition (**Table 4**) observed for the DRVYVYPβF analog. Notable decreases in ACE2 inhibition were also observed for β-Pro and β-Phe substitutions in native Ang II in a previous study (Clayton et al., 2011). Native Ang II and chimeric analogs showing high levels of ACE2 binding at 10 μM were then assessed at reduced concentration, first at 1 μM and then at 0.1 μM if measurable inhibition was observed. The two non-β-amino acid analogs, DRVYIYPF and DRVYVYPF, retained significant levels of ACE2 binding when assessed at lower concentration with 17 and 31% inhibition observed at 0.1 μM [**Table 4** – native AngII, 21% Inh. at 1 μm, no (–5%) Inh. at 0.1 μM]. Analogs that showed notable reductions in apparent ACE2 binding (**Table 2**, inhibition data) were assayed at an increased concentration of 100 μM in order to adequately assess ACE2 proteolytic stability.

All Ang II chimeras showed increased ACE2 proteolytic stability when assessed by RP–HPLC, though with reductions to ACE2 binding also observed for several peptides. DRVYIYPF showed 96% inhibition at 10 μM and 18% cleavage over 5 h as compared to 40% cleavage of native Ang II (**Table 4**). DRVYVYPF showed even more enhanced proteolytic stability with approximately 4-fold less (9%) cleavage by ACE2 over 5 h compared to native Ang II (40%). β-Pro and β^~^-Phe Ang II analogs, DRVYIYβPF, DRVYVYβPF, DRVYIYPβF, and DRVYVYPβF showed complete resistance to proteolytic cleavage at a concentration where substantial ACE2 binding occurred (**Table 4**, 100 μm cleavage data). DRVYVYβPF is the AngII analog showing the highest levels of ACE2 binding (42%, 10 μM) and showing complete proteolytic stability to ACE2.

### SECONDARY STRUCTURE DETERMINED BY CD

**FIGURE 1:**
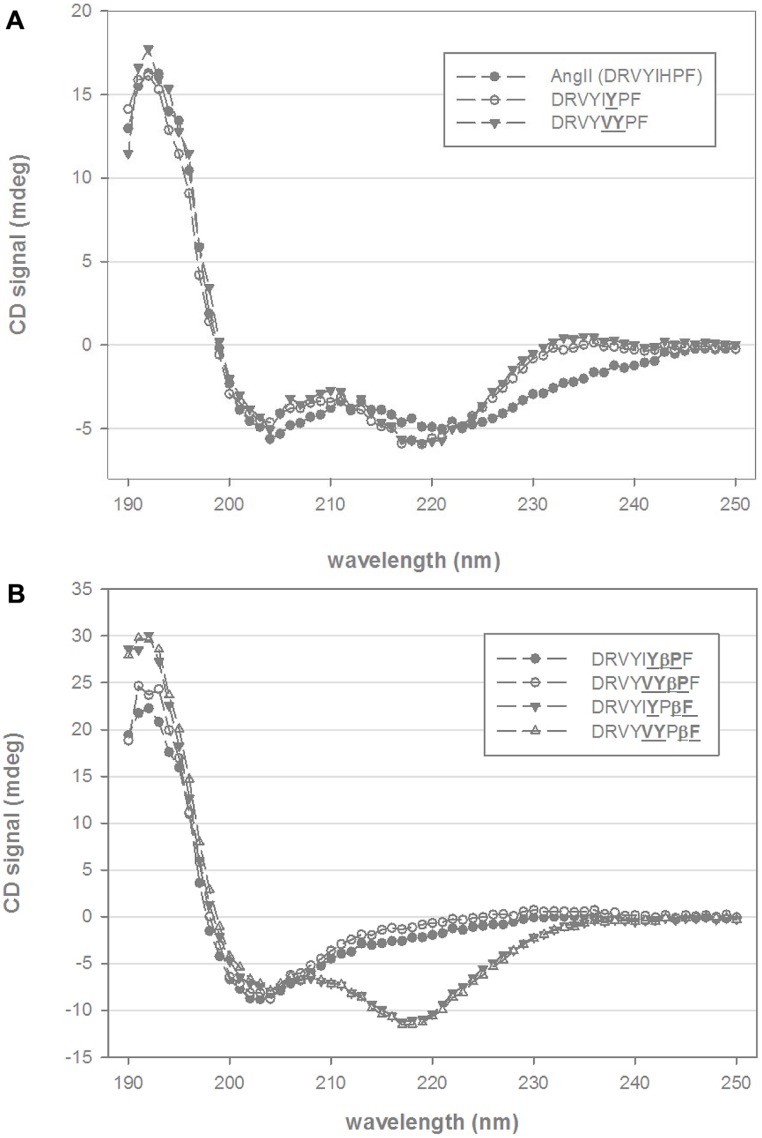
**Circular dichroism (CD) spectra of chimeric AngII analogs.** **(A)** Shows the CD spectra of native AngII and two C-terminal non-β-amino acid analogs (DRVYIYPF, DRVYVYPF) in 25% trifluoroethanol (TFE) and 10 mM phosphate buffer. **(B)** Shows four β-amino acid analogs (βPro and βPhe, scissile-bond is between Pro and Phe residues AngII sequence) of DRVYIYPF and DRVYVYPF in similar mixtures of TFE and aqueous phosphate buffer.

Circular dichroism spectra of native Ang II in mixtures of TFE and water at pH 7.0 indicate elements of both β-turn and α-helical structure with minima between 205 and 220 nm and with a stronger absorbing CD component around 190 nm (**Figure 1A**). A broad CD signal present between 220 and 245 nm is consistent with the presence of mixed β-sheet and β-turn conformation. Substitution of the histidine residue by tyrosine (DRVYIYPF) and further substitution of isoleucine to valine (DRVYVYPF) resulted in a slightly more pronounced 220 nm minima and the disappearance of the broad signal at 230–240 nm, suggesting a loss of the β-sheet and β-turn components and more pronounced helical conformation (**Figure 1A**). Inclusion of a β-proline, an extension to the peptide backbone by one methylene group, to the second residue from the C-terminal for either of these analogs (DRVYIYβPF and DRVYVYβPF) resulted in structural changes with a notable decrease in the helical content as indicated by the loss of CD signal at ∼220 nm. (**Figure 1B**). As the maximum at 190–195 nm is still present, it is likely that a significant amount of stabilized secondary structure is still present (such as a mixture of β-sheet or β-turn structure particularly given the red-shifted random coil minima and a broad CD signal in the observed 205–220 nm wavelength range) though further analysis is required given the complex spectra that can arise from mixtures of secondary structure. Notably different structural changes were observed when a β-amino acid was introduced at the C-terminal phenylalanine for DRVYIYPβF and DRVYVYPβF; **Figure 1B**) with distinct minima at 217/218 nm and maxima at 190–195 nm suggesting α-helical structure together with a possible β-sheet-like component (Greenfield, 2006a,b).

### ANGIOTENSIN RECEPTOR BINDING

The chimeric Ang II peptides and their β-analogs were screened for binding to AT_1_ and AT_2_ receptors (AT_1_R and AT_2_R) up to saturating concentration to determine the relative receptor selectivity. The results are listed in **Table 5** and revealed different binding properties of these peptides for each receptor, with all but one analog showing dramatic losses in binding to the AT_1_R while AT_2_R binding varied in the range from 25% to a value similar to that of native Ang II. Substitution of histidine for tyrosine and/or isoleucine for valine resulted in complete loss of AT_1_R binding and this was also seen for both the β-Pro analogs of these peptides. Of the two β-Phe analogs, the double substitution analog (DRVYVYPβF) showed no AT_1_R binding, while the single substitution analog showed ∼30% binding compared to native Ang II (**Table 5**). AT_2_R binding requirements were shown to be less stringent than AT_1_R binding as all Ang II analogs had some level of AT_2_R binding, with both the single and double non-β-amino acid substitutions exhibiting similar binding as native Ang II. Small decreases in AT_2_R binding were observed for both of the β-Pro analogs, with 80 and 75% binding observed for the single and double substitution analogs, respectively. Substantial decreases in binding were seen when the C-terminal Phe residue was replaced with its β-analog with reductions to receptor binding of ∼75% observed for both single and double substitution peptides (**Table 5**).

**Table 5:** Relative binding of various Ang II analogs to AT_**1**_R and AT_**2**_R.

Peptide sequence	AT1Rbinding (%)^a^	AT2Rbinding (%)^a^
D_1_R_2_V_3_Y_4_I_5_H_6_P_7_F_8_	>95	>95
D_1_R_2_V_3_Y_4_I_5_**Y**_6_P_7_F_8_	0	>95
D_1_R_2_V_3_Y_4_**V**_5_**Y**_6_P_7_F_8_	0	>95
D_1_R_2_V_3_Y_4_I_5_**Y_6_βP_7_**F_8_	0	80
D_1_R_2_V_3_Y_4_**V_5_Y_6_βP_7_**F_8_	0	75
D_1_R_2_V_3_Y_4_I_5_**Y**_6_P_7_**βF_8_**	30	25
D_1_R_2_V_3_Y_4_**V**_5_**Y**_6_P_7_**βF_8_**	0	25

*Unmodified Angiotensin II sequence – H_2_N- 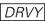 - 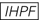 -COO(-)*.

*^a^AT receptor binding shown as compared to native AngII binding (which represents 100 displacement) concentration of ^*14*^C labeled receptor binding peptide. binding as determined at 1 μM concentration*.

## DISCUSSION

The conventional wisdom that maximum blockage of RAS results in the best therapeutic outcome is now considered an over-simplistic model, and the molecular detail of key binding partners in RAS is also poorly understood. Thus, in spite of the large number of anti-hypertensive agents on the market, new therapeutic approaches are always required to meet the challenges of personalized medicine with minimal side-effects. A more detailed description of the molecular binding interactions and RAS ligand binding specificities will facilitate understanding this complex system and facilitate drug design.

Angiotensin converting enzyme 2, AT_1_R and AT_2_R all play a central role in this constantly evolving scenario and our studies provide new insight into the structure and function of these proteins. We have investigated the topographical and structural requirements for the binding of the C-terminal region of Ang II to ACE2, AT_1_R, and AT_2_R. We employed a focused library approach to characterize the binding determinants in the Ang II C-terminal tetrapeptide template IHPF. The results identified four substitutions that enhanced apparent binding to ACE2. A series of seven tetrapeptide analogs was then generated which all exhibited significant binding at 10 μM. Apparent binding data revealed that Val and Pro at position 1, Tyr in position 2, and Val in position 3 of IHPF yielded maximum binding. N-terminal acetylation increased the apparent binding for the parent IHPF and two of the analogs (IYPF and VYPF), while C-terminal amidation reduced the binding of all peptides except VHVF. Combined N-terminal acetylation and C-terminal amidation significantly reduced apparent binding further demonstrating the requirement for the C-terminal carboxyl group for ACE2 binding.

IYPF and VYPF were subsequently used to generate a series of Ang II octapeptide analogs to further explore the relative binding requirements to ACE2, AT_1_R, and AT_2_R. Specifically, these two tetrapeptides were grafted onto the N-terminus of Ang II to yield a range of chimeric Ang II analogs. In addition, β-amino acid analogs were also synthesized to exploit the selectivity and proteolytic stability effects previously observed (Clayton et al., 2011; Jones et al., 2011). The results are summarized in **Table 6** and reveal a ladder of selectivity changes within the series of six peptides. Relative to Ang II, peptides with Tyr^6^ or Tyr^6^/Val^5^ both lost AT_1_R binding but maintained ACE2 and AT_2_R binding and also both exhibited increased helical structure. These results agree with our previous study (Clayton et al., 2011; Jones et al., 2011) and others (Magnani et al., 2014) who also observed that changes at residue 6 in Ang II lead to AT_2_R-selective ligands. The present results also further demonstrate that a bulky hydrophobic residue is equally tolerated by both ACE2 and AT_2_R. By comparison, when Pro^7^ was replaced by β-Pro^7^ in both of these peptides, there was a substantial loss in ACE2 binding but AT_2_R binding was largely maintained. This change was also accompanied by a loss in apparent helicity suggesting that the selectivity between ACE2, AT_1_R, and AT_2_R can be controlled by both the physicochemical properties of specific amino acids in Ang II and also manipulation of the conformational properties of the peptide. Finally, substitution of Phe^8^ with β-Phe^8^ resulted in a loss of ACE2 binding and a further substantial decrease in AT_2_R binding and the adoption of a different secondary structure. Binding of Ang II analogs to the AT_2_R was therefore less dependent on secondary structure and/or amino acid side chain presentation compared to AT_1_R binding. AT_1_R binding was therefore more sensitive to structure and/or side chain presentation as all but one Ang II analog showed minimal AT_1_R binding and is consistent with previous studies (Miura and Karnik, 1999; Rosenstrom et al., 2004a).

**Table 6:** Overview of the different AngII analogs, structural changes, and altered binding specificities^**a,b,c**^.

Peptide sequence^a^	CD spectral changes	Structural content/change relative to Ang II	Apparent inhibition (%)^b^	Cleavage (%)^a,b^	AT_1_R binding (%)^c^	AT_2_R binding (%)^c^
			10 μM	10 μM	100 nM	100 nM
D_1_R_2_V_3_Y_4_I_5_H_6_P_7_F_8_	Maximum 194 nm, minimum 208, 222 nm	Control, native Angiotensin II(β- turn/helical)	77 ± 2.9	40 ± 2	>95	>95
D_1_R_2_V_3_Y_4_I_5_**Y**_6_P_7_F_8_	More pronounced minima 205, 220 nm reduced at 230–245 nm	Increased helicity	96 ± 0	18 ± 1.2	0	>95
D_1_R_2_V_3_Y_4_**V**_5_**Y**_6_P_7_F_8_	More pronounced minima 205, 220 nm reduced at 230–245 nm	Increased helicity	98 ± 0.3	9 ± 0.3	0	>95
D_1_R_2_V_3_Y_4_I_5_**Y_6_*β*P_7_**F_8_	Large decrease 220 nm increase in 205 nm peak, shift to ∼200 nm	Loss of helicity	10 ± 5.8	0 ± 0	0	80
D_1_R_2_V_3_Y_4_**V_5_Y_6_*β*P_7_**F_8_	Large decrease 220 nm increase in 205 nm peak, shift to ∼200 nm	Loss of helicity	42 ± 4.4	0 ± 0	0	75
D_1_R_2_V_3_Y_4_I_5_**Y**_6_P_7_***β*F_8_**	Increase at 195 nm notable increase at 217 nm	Mixed helical and β-sheet/turn content	0 ± 5	0 ± 0	30	25
D_1_R_2_V_3_Y_4_**V**_5_**Y**_6_P_7_***β*F_8_**	Increase at 195 nm notable increase at 217 nm	Mixed helix and β-sheet/turn content	15 ± 9	0 ± 0	0	25

^a^ACE2 cleaves AngII between P7 and F8; ^b^data shown as ‘average ± standard error’; ^c^AT receptor binding shown as % as compared to native AngII binding (which represents 100% displacement) concentration of ^*14*^C labeled receptor binding peptide.

It is now clear that the central to C-terminal region of Ang II can be modified to engineer selectivity of binding to different Ang II binding partners. Our present results are consistent with our previous findings where single β-amino acid substitutions to Ang II resulted in Ang II analogs which conferred marked selectivity for agonism at AT_2_R over AT_1_R (Jones et al., 2011). Our findings are also consistent with other studies that demonstrated the importance of the central region of Ang II for AT_2_R binding. For example, substitution of His^6^ by 4-NH_2_-Phe^6^ in Ang II produced a peptide with high AT_2_R-to-AT_1_R selectivity (Speth and Kim, 1990; Rosenstrom et al., 2004a,b). Others have also shown that modifications to the Tyr^4^-Ile^5^ residues of Ang II result in AT_2_R-selective compounds (Johannesson et al., 2004; Rosenstrom et al., 2004b). Finally, Tyr^6^-Ang II has also been shown to bind selectively to AT_2_R (Magnani et al., 2014). A general strategy to design AT_1_R-, or AT_2_R-selective ligands for therapeutic application is to use the key apolar, cyclic and imidazolic binding functionalities of the central and C-terminal of Ang II as a template for generating small molecules libraries to be screened for selective AT receptor binding activity (Agelis et al., 2012, 2013; Sharma, 2014; Veron et al., 2014). Other approaches exploit the conformational aspects of Ang II-AT receptor interaction to design selective ligands (Oliveira et al., 2011). Our results provide new information on the structural determinants for optimal amino acid residues and functional group combinations for RAS-ligand binding to inform future peptidomimetic design for both of these design approaches.

Overall, the binding and stability profile of these stabilized peptides may modulate hypertensive profile by either providing a stable Ang II analog that could act on the AT_2_R without inactivation or by inhibiting ACE2 and so prolonging the action of endogenously produced Ang II. Functional assays on these Ang II peptidomimetics are therefore required to indicate agonist or antagonist activity, together with *in vivo* studies to profile the action of these compounds and to elucidate the therapeutic potential of these compounds, given that cardiovascular tone is controlled by the action of several proteins including ACE, ACE2, AT_1_R, and AT_2_R.

## CONCLUSION

The last decade has seen the discovery of several new components of the RAS which is now seen as a balance between the pro-vasoconstrictor, pro-fibrotic, pro-growth axis and the pro-vasodilatory, anti-fibrotic, anti-growth arm. Hypertension is one of the cardiovascular diseases that may cause cardiovascular remodeling and endothelial dysfunction on top of high blood pressure. ACE2, AT_1_R, and AT_2_R all play a central role in this constantly evolving scenario and our studies provide new insight into the structure and function of these proteins. In particular, we have investigated the topographical and structural requirements for the binding of the C-terminal region of Ang II to ACE2, AT_1_R, and AT_2_R. We employed a focused library approach to characterize the binding determinants in the Ang II C-terminal tetrapeptide template IHPF and the results identified four substitutions that enhanced apparent binding to ACE2. The Ang II chimeras identified in this study revealed key residues, side chain functionalities and structure-binding relationships which can be used to inform a small molecule drug design approach for more specific and selective control cardiovascular function. As such, this type of peptidomimetic design shows great potential for the production of research tools to provide insight into the structure and function of key members of RAS.

## Conflict of Interest Statement

The authors declare that the research was conducted in the absence of any commercial or financial relationships that could be construed as a potential conflict of interest.

## ABBREVIATIONS

ACE2: angiotensin converting enzyme 2
Ang II: angiotensin II
AT1R: angiotensin II type 1 receptor
AT2R: angiotensin II type 2 receptor
LC: liquid chromatography
MS: mass spectrometry
QFS: quenched fluorescence substrate
RAS: renin–angiotensin system
